# GTZ: a fast compression and cloud transmission tool optimized for FASTQ files

**DOI:** 10.1186/s12859-017-1973-5

**Published:** 2017-12-28

**Authors:** Yuting Xing, Gen Li, Zhenguo Wang, Bolun Feng, Zhuo Song, Chengkun Wu

**Affiliations:** 1 School of Computer Science, National University of Defense Technology, Changsha, 410000 China; 2 Genetalks Biotech. Co.,Ltd., Beijing, 100000 China

**Keywords:** FASTQ, Compression, General-purpose, Lossless, Parallel compression and transmission, Cloud computing

## Abstract

**Background:**

The dramatic development of DNA sequencing technology is generating real big data, craving for more storage and bandwidth. To speed up data sharing and bring data to computing resource faster and cheaper, it is necessary to develop a compression tool than can support efficient compression and transmission of sequencing data onto the cloud storage.

**Results:**

This paper presents GTZ, a compression and transmission tool, optimized for FASTQ files. As a reference-free lossless FASTQ compressor, GTZ treats different lines of FASTQ separately, utilizes adaptive context modelling to estimate their characteristic probabilities, and compresses data blocks with arithmetic coding. GTZ can also be used to compress multiple files or directories at once. Furthermore, as a tool to be used in the cloud computing era, it is capable of saving compressed data locally or transmitting data directly into cloud by choice. We evaluated the performance of GTZ on some diverse FASTQ benchmarks. Results show that in most cases, it outperforms many other tools in terms of the compression ratio, speed and stability.

**Conclusions:**

GTZ is a tool that enables efficient lossless FASTQ data compression and simultaneous data transmission onto to cloud. It emerges as a useful tool for NGS data storage and transmission in the cloud environment. GTZ is freely available online at: https://github.com/Genetalks/gtz.

**Electronic supplementary material:**

The online version of this article (10.1186/s12859-017-1973-5) contains supplementary material, which is available to authorized users.

## Background

Next generation sequencing (NGS) has greatly facilitated the development of genome analyses, which is vital for reaching the goal of precision medicine. Yet the exponential growth of accumulated sequencing data poses serious challenges to the transmission and storage of NGS data. Efficient compression methods provide the possibility to address this increasingly prominent problem.

Previously, general-propose compression tools, such as gzip (http://www.gzip.org/), bzip2 (http://www.bzip.org/) and 7z (www.7-zip.org), have been utilized to compress NGS data. These tools do not take advantage of the characteristics of genome data, such as a small size alphabet and repeated sequences segments, which leaves space for performance optimization. Recently, some specialized compression tools have been developed for NGS data. These tools are either reference-based or reference-free. The main difference lies in whether extra genome sequences are used as references. Reference-based algorithms encode the differences between the target and reference sequences, and consume more memory to improve compression performance. GenCompress [1] and SimGene [2] use various entropy encoders, such as arithmetic, Golomb and Huffman to compress integer values. The values show properties of reads, like starting position, length of reads, etc. A statistical compression method, GReEn [3], uses an adaptive model to estimate probabilities based on the frequencies of characters. The probabilities are then compressed with an arithmetic encoder. QUIP [4] exploits arithmetic coding associated with models of order-3 and high-order Markov chains in all three parts of FASTQ data. LW-FQZip [5] utilized incremental and run-length-limited encoding schemes to compress the metadata and quality scores, respectively. Reads are pre-processed by a light-weight mapping model and then three components are combined to be compressed by a general-purpose tool, like LZMA. Fqzcomp [6] estimates character probabilities by order-k context modelling and compresses NGS data in FASTQ format with the help of arithmetic coders.

Nevertheless, reference-based algorithms can be inefficient if the similarity between target and reference sequences is low. Therefore, reference-free methods were also proposed to address this problem. Biocompress proposed in [7] is a compression method dedicated to genomic sequences. Its main idea is based on the classical dictionary-based compression method --the Ziv and Lempel [8] compression algorithm. Repeats and palindromes are encoded using the length and the position of their earliest occurrences. As an extension of biocompress [7], biocompress-2 [9] exploits the same scheme, and uses arithmetic coding of order-2 when no significant repetition exists. The DSRC [10] algorithm splits sequences into blocks and compresses them independently with LZ77 [8] and Huffman [11] encoding. It is faster than QUIP both in compression and decompression speed, but inferior to the later in terms of compression ratio. DSRC2 [12], the multithreaded version of DSRC [10], splits the input into three streams for pre-processing. After pre-processing, metadata, reads, and quality scores are compressed separately in DRSC. A boosting algorithm, SCALCE [13], which re-organizes the reads, can outperform other algorithms on most datasets both in the compression ratio and the compression speed.

Nowadays, it is evident that cloud computing has become increasingly important for genomic analyses. However, above-mentioned tools were developed for local usage. Compression has to be completed locally before a data transmission onto the cloud can begin.

AdOC proposed in [14] is a general-propose tool that allows the overlap of compression and communication in the context of a distributed computing environment. It presents a model for transport level compression with dynamic compression level adaptation, which can be used in an environment where resource availability and bandwidth vary unpredictably.

Generally, the compression performances of the universal compression algorithms, such as AdOC, are unsatisfactory for NGS datasets.

In this paper, we present a tool GTZ, it is characterized as a lossless and efficient compression tool to be used jointly with cloud computing for large-scale genomic data analyses:

1. GTZ exploits context model technology combined with multiple prediction modelling schemes. It employs paralleling processing to improve the compression speed.
2. GTZ can compress directories or folders into a single archive, which is called a multi stream file system. The all-in-one scheme can satisfy purposes of transmission, validation and storage.
3. GTZ supports random access to files or archives. GTZ utilizes block storage, such that users can extract some parts of genome sequences out of a FASTQ file or some files in a folder, without a complete decompression of the compressed archive.
4. GTZ can transfer compressed blocks to the cloud storage while the compress is still in process, which is a novel feature compared with other compression tools. This feature enables the data transmission time to be can greatly reduce the total time needed for compression and data transmission onto the cloud. For instance, it could compress and transit a 200GB FASTQ file to cloud storages like AWS and Alibaba cloud storage within 14 min.
5. GTZ provides a Python API, through which users can integrate GTZ in their own applications flexibly.

In the remaining of this paper, we will introduce how GTZ works and evaluate its performance on several benchmark datasets using the AWS service.

## Methods

**Fig. 1:**
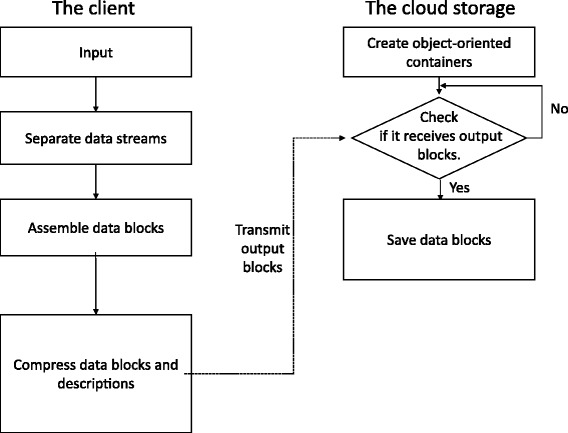
The workflow of GTZ

GTZ supports efficient compression in parallel, parallel transmission and random fetching. Figure 1 demonstrates the workflow of GTZ processing.

GTZ involves procedures on clients and the cloud end.

A client takes the following steps:

1. Read in streams of large data files.
2. Pre-process the input by dividing data streams into three sub-streams: metadata, base sequence, and quality score.
3. Buffer sub-streams in local memories and assemble them into different types of data blocks with a fixed size.
4. Compress assembled data blocks and their descriptions, and then transmit output blocks into the cloud storage.

**Fig. 2:**
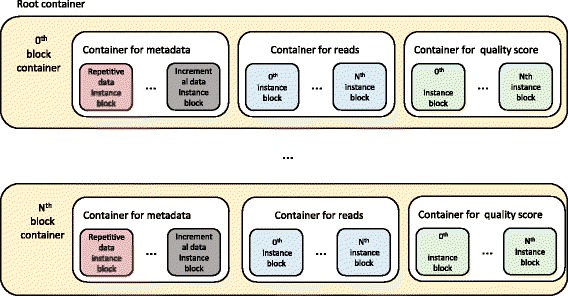
The hierarchy of data containers

On the cloud, the followings steps are executed:

1. Create three types of object-oriented containers (shown in Fig. 2), which define a tree structure.
2. Loop and wait to receive output blocks sent by the client.
3. Save received output blocks into block containers according to their types.
4. Stop if no more output blocks are received.

We will explain all the steps in further details about processing FASTQ files below:

### The client reading streams of large data files

Raw NGS data files are typically stored in FASTQ format for the convenience of compression. A typical FASTQ file contains four lines per sequence: Line 1 begins with a character ‘@’ followed by a sequence identifier; Line 2 holds the raw sequence composed of A, C, T, and G; line 3 begins with a character ‘+’ and is optionally followed by the same sequence identifier (and any description) again; line 4 holds the corresponding quality scores in ASCII characters for the sequence characters in line 2. An example of a read is given in Table 1.

**Table 1:** The format of an FASTQ file

1	@ERR194147.1.HSQ1004:134:C0D8DACXX:1:1104:3874:86,238/1
2	GGTTCCTACTTNAGGGTCATTAAATAGCCCACACGTC
3	+
4	CC@FFFFFHHH#JJJFHIIJJJJJJJIJHIJJJJJJJ

### Data pre-processing

**Fig. 3:**
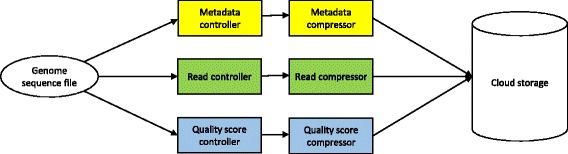
Pre-process data files with pre-processing controllers and compression units

During the second step, a data stream is split into metadata sub-streams, base sequence sub-streams and quality score sub-streams. (Since uninformative comment lines normally do not provide any useful information for compression, comment streams are omitted during pre-processing.) Three types of date pre-processing controllers buffer sub-streams and save them in data blocks at a fixed size respectively. Afterwards, data blocks with annotations (about numbers of blocks, sizes of blocks and types of streams) are sent to corresponding compression units. Figure 3 demonstrates how to pre-process data files with the help of pre-processing controllers and compression units.

### Compressing data

GTZ is a general-purpose compression tool that uses statistical modelling (http://marknelson.us/1991/02/01/arithmetic-coding-statistical-modeling-data-compression/) and arithmetic coding.

Statistical modelling can be categorized into two types: static and adaptive statistical modelling. Conventional methods are normally static, which means probabilities are calculated after sequences are scanned from the beginning to end. A static modelling keeps a static table that records character-frequency counts. Although they produce relatively accurate results, the drawbacks are obvious:

1. It is time-consuming to read all the sequences into main memory before compression.
2. If an input stream does not match well with the previously accumulated sequence, the compression ratio will be degraded, even the output stream will become larger than the input stream.

In GTZ, we employ an adaptive statistical data compression technique based on context modelling. An adaptive modeling needs not to scan the whole sequence and generate probabilities before coding. Instead, the adaptive prediction technology provides on-the-fly reading and compression, that is probabilities are calculated based on the characters already read into the memory. Probabilities may alter with more characters scanned. Initially, the performance of adaptive statistical modelling may be poor due to the lack of reads. However, with more sequences processed, the prediction tends to be more accurate.

Every time the compressor encodes a character, it will update the counter in the prediction table. When a new character X (suppose the sequence before X is ABCD) comes, GTZ will traverse the prediction table, find every character that has followed ABCD before, and compare their appearance frequencies. For instance, if both ABCDX appears 10 times, and ABCDY only once. Then GTZ will assign a higher probability for X.

**Fig. 4:**
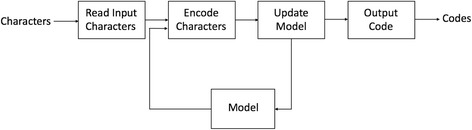
Work flow of a typical statistical modelling

The work flow of an adaptive model is depicted in Fig. 4. The box ‘Update model’ means converting low-order modellings to high-order modellings (the meaning of low-order and high-order will be discussed in the next subsection.).

Adaptive prediction modelling can effectively reduce compression time. There is no need to read all sequences in a time and it introduces overlap of scanning and compression.

GTZ utilizes specific compression units for different kinds of data blocks: a low-order encoder for genetic sequences, a multi-order encoder for quality scores and mixed encoders for metadata. Finally, the outputs in this procedure are blocks at a fixed size.

The main idea about arithmetic coding is to convert reads into a floating point ranging from zero to one (precisely greater than or equal to zero and less than one) based on the predictive probabilities of characters. If the statistical modelling estimates every single character accurately for the compressor, we will have high compression performance. On the contrary, a poor prediction may result in expansion of the original sequence, instead of compression. Thus, the performance of a compressor largely relies on the whether the statistical modelling can output near-optimal predictive probabilities.

### A low-order encoder for reads

The simplest implementation of adaptive modeling is order-0. Exactly, it does not consider any context information, thus this short-sighted modeling can only see the current character and make prediction that is independent of the previous sequences. Similarly, an order-1 encoder makes prediction based on one preceding character. Consequently, the low-order modeling makes little contribution to the performance of compressors. Its main advantage is that it is very memory efficient. Hence, for quality score streams that do not have spatial locality, a low-order modeling is adequate for moderate compression rate.

**Fig. 5:**
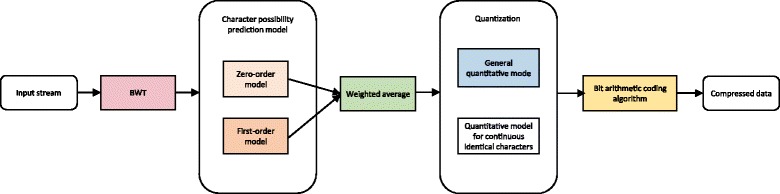
A low-order encoder scheme

Our tailored low-order encoder for reads is demonstrated in Fig. 5. The first step is to transform sequences with the BWT algorithm. BWT (Burrows-Wheeler transform) rearranges reads into runs of similar characters. In the second step, the zero-order and the first-order prediction model are used to calculate appearance probability of each character. Since a poor probability accuracy contributes to undesirable encoding results, we add interpolation after quantizing the weighted average probability, to reduce prediction errors and improve compression ratios. In the last procedure, the bit arithmetic coding algorithm produces decimals ranging from zero to one as outputs to represent sequences.

### A multi-order encoder for quality scores

The statistical modeling needs non-uniform probability distribution for arithmetic algorithms. The high-order modeling enables high probabilities for those characters which appear frequently, and low probabilities for those which appear infrequently. As a result, compared with low-order encoders, higher-order encoders can enhance adaptive modeling.

A high-order modeling considers several characters preceding the current position. It can obtain better compression performance at the expense of more memory usage. Higher-order modeling was less used due to the limited memory capacity, which is no longer a problem anymore.

**Fig. 6:**
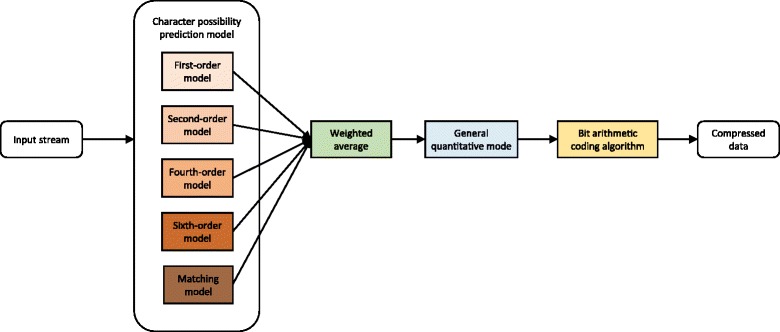
A multi-order encoder scheme

Without transformation, a multi-order encoder (See Fig. 6) for quality scores includes two procedures:

Firstly, to generate probabilities of characters, input stream flows through an expanding character probability prediction model, which is composed of first-order, second-order, fourth-order, sixth-order prediction models and a matching model. Like a low-order encoder, probabilities of characters undergo weighted averaging, quantization and interpolation to obtain final results. Secondly, we use bit arithmetic coding algorithm for compression.

### A hybrid scheme for metadata

For metadata sub-streams, GTZ first uses delimiters (punctuations) to split them into different segments, then uses different ways to process metadata according to their fields:

For numbers in an ascending or descending order, we employ incremental encoding to represent the variations of one metadata to its preceding neighbors. For instance, ‘3458644’ will be compressed into 3,1,1,3,-2,-2,0. For continuous identical characters, we exploit run-length limited encoding to show their values and numbers of repetition. For random numbers with various precisions, we convert their formats by UTF-8 coding without adding a single separator, and then use a low-order encoder for compression. Otherwise, use the low-order encoder to compress metadata.

In conclusion, during this process, sub-streams are fed into a dynamic probability prediction model and an arithmetic encoder, and they are transformed into compressed blocks at a fixed size.

### Data transmission

The key objective is to transmit output blocks to a certain cloud storage platform, with annotations about types, sizes, numbers of data blocks.

To note, different types of encoders may lead to inconsistency in compression speed, which can lead to a data pipe blockage. Thus, in our system, the pipe-filter pattern is designed to synchronize input and output speed, e.g., the input flow will be blocked when the speed of input stream is faster than that of the output stream; The pipe will also be blocked when there is no input flow.

### Storage at the cloud end — Creating an object-oriented nested container system

GTZ creates containers as storage compartments that provide a way to manage instances and store file directories. They are organized in a tree structure. Containers can be nested to represent locations of instances: a root container represents a complete compressed file; a block container includes different types of sub-stream containers where specific instances are stored. The nesting structure is showed in Fig. 2.

A root container represents a FASTQ file and it holds N block containers, each of which includes metadata sub-containers, base sequence sub-containers and quality score sub-containers. A metadata sub-container nests repetitive data blocks, random data blocks, incremental data blocks, etc. Base sequence sub-containers and quality score sub-containers nest 0 instance block to N instance block. Taking base sequences for examples, the 0 to (N-1) output blocks are stored in the 0th block container, and the N to (2 N-1) output blocks are stored in the 1st block container, and so on.

This kind of hierarchy allows users to maintain a directory structure to manage compressed files, thereby facilitating random access to specific sequence. Here, we show how to decompress and extract the target files from the compressed archive: in decompression mode, the system will index the start line number *n* (which is given by users through the command line), then fetch the certain sequence from their according block containers and compress certain (which are also specified by users) lines of the sequence.

### Receive data — Receive and store output blocks

Cloud storage platform receives output blocks and descriptive information such as numbers of data blocks, sizes of data blocks, most importantly, the line number of every base sequence within data blocks. The description enables us to directly index certain sequences with line numbers and decode their affiliated blocks rather than extract the whole file. Output blocks are stored in corresponding types of containers.

What is worth noting is that non-FASTQ files can also be compressed and transmitted through GTZ. Additionally, GTZ uses object-oriented programming, it is not restricted to interact with a specific type of cloud storage platform, but applicable to most existing cloud storage platforms, such as the Amazon Web Service and the Alibaba cloud.

## Results and discussion

In this section, we conducted experiments on a 32-core AWS R4.8xlarge instance with 244GB of memory to evaluate the performance of GTZ in terms of compression ratio and compression speed. During the experiments, the following points should be noted:

1. Considering that our method is lossless, we exclude methods that allow losses as counterparts.
2. NGS data can be stored in either FASTQ or SAM/BAM formats, we only take into account tools targeted at FASTQ format files.
3. Comparison will be conducted among the algorithms that do not reorder input sequences.

We carried out tests on 8 publicly accessible FASTQ datasets, which are downloaded from the Sequence Read Archive(SRA) initiated by NCBI and the GCTA competition website (https://tianchi.aliyun.com/mini/challenge.htm#training-profile). To ensure the comprehensiveness of our evaluation, we chose datasets that are heterogeneous: the size of datasets ranges from 556MBs to 202, 631MBs; different species and different types of data were chosen, including DNA reads, one RNA-seq dataset of *Homo sapiens*, one metagenome dataset and read 2 of NA12878 (the GCTA competition datasets). Different quality score encoding methods, such as Sanger and Illumina 1.8+, are selected to cover different numbers of quality scores in datasets. Quality scores are logarithmically linked to error probabilities, leading to a larger alphabet than meta data and reads, thus encodings with small numbers of quality scores normally contribute to a higher compression performance. Descriptions of the datasets are listed in Table 2. Besides, for comparison, based on a comprehensive literature survey, we selected four state-of-the-art and widely-used lossless compression algorithms, including DSRC2 [12] (the improved version of DSRC [10]), quip [4], LW-FQZip [5], Fqzcomp [6], LFQC [15] and pigz. Among them, LW-FQZip [5], Fqzcomp [15] are representatives of reference-based tools; DSRC2 [12] and quip [4] are reference-free methods; pigz is a general-purpose tool for compression. All the experimental results are included in Additional file 1.

**Table 2:** Descriptions of 8 FASTQ datasets used for performance evaluation

Dataset	Species	Reference genome size	Encoding	No. of quality scores in data file
ERR233152	*P. aeruginosa*	556	Sanger	32
SRR935126	*A. thaliana*	9755	Sanger	39
SRR489793	*C. elegans*	12,807	Illumina 1.8+	38
SRR801793	L. pneumophila	2756	Sanger	38
SRR125858	*H. sapiens*	50,744	Sanger	39
SRR5419422	RNA seq (H. sapiens)	15,095	Illumina 1.8+	6
ERR1137269	metagenomes	56,543	Illumina 1.8+	7
NA12878 (read 2)	H. sapiens	202,631	Sanger	38

### Evaluation results

We evaluated the performance of different tools by the following related metrics: the compression ratio, the coefficient of variation (CV) of compression ratios, the compression speed, the total time of compression and transmission to cloud storages. Specifically, the compression ratio is defined as follows:

According to this definition, a smaller compression ratio represents a more effective compression in terms of size reduction; The coefficient of variation (CV) stands for the extent of variability in relation to the mean and it is defined as the ratio of the standard deviation (SD) divided by the average (avg):

A smaller CV reveals better robustness and stability; additionally, GTZ not only performs well in compression on local computers, but also gains satisfactory results in transmission to cloud storages. On local computers, the compression speed is chosen for evaluation, and it can be simply measured by the time used for the compression (for different tools applied on the same data). Under the latter circumstance, the run time of algorithms should be the sum of compression and transmission time, namely, from the start of compression to the completion of transmission onto the cloud.

### Compression ratio

**Fig. 7:**
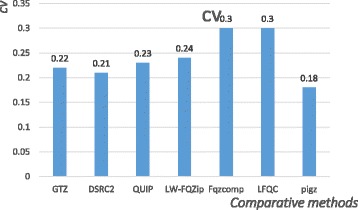
CVs for the compression ratio of different tools

Performance evaluation results are demonstrated in Table 3 and the best compression ratio, the best CV, which are the smallest, are boldfaced. Comparative results of CV are shown in Fig. 7.

**Table 3:** Compression ratios of different tools on 8 FASTQ datasets

Dataset	Compression ratio (%)						
	GTZ	DSRC2	QUIP	LW-FQZip	Fqzcomp	LFQC	pigz
ERR233152	15.9	16.7	19	19	16.8	**8**	26.4
SRR935126	18.6	19.6	17.7	20.5	17.8	**9.9**	30.2
SRR489793	22.8	22.7	22.6	25.5	22.5	**12.8**	34.4
SRR801793	21.4	21.9	21.1	21.2	20.8	**12.3**	34.1
SRR125858	19.4	19.5	18.9	23.1	28.9	**17.6**	31
SRR5419422	12.8	13.9	**10.9**	12.5	12	ERROR	22
ERR1137269	12.2	13.4	12.8	14.3	**11.9**	ERROR	21.9
NA12878 (read 2)	**19.8**	24	20.4	TLE	19.9	TLE	24.7
avg	17.86	18.96	17.93	19.44	18.83	**12.12**	28.09
SD	3.87	3.97	4.07	4.64	5.60	3.62	5.05
CV	0.22	0.21	0.23	0.24	0.30	0.30	**0.18**

The best results of all the tools are boldfaced

To note, in Table 3, some fields on datasets NA12878 (read 2, a very large dataset) are filled with “TLE” (Time Limit Exceeded, the threshold is empirically set as 6 h), and some fields of the LFQC tools on the SRR5419422, ERR137269 datasets are filled with “Error” (Cannot decompress after compression, those two datasets represent RNA sequences and metagenomics data respectively). Those “outliers” represent a low robustness (for convenience of CV calculation, we just filter out “TLE” and “Error”). For instance, LFQC [15] yields the best result on 5 out of 8 datasets. However, it got “TLE” on three datasets, which means a poor stability in compression efficiency. In addition, despite the CV of pigz is the lowest, its average compression ratio ranks at the bottom. Moreover, GTZ ranks second with an average compression ratio of 17.86%, and the CV of GTZ is far below that of LFQC [15] (which has the best compression ratio). In summary, GTZ not only maintains a relatively good average compression ratio than most of its counterparts, but also exhibits better stability and robustness when dealing with different datasets.

### Compression speed

Results for the compression speed tests are shown in Table 4 and the best results are boldfaced. LFQC [15] and LW-FQZip [5] fail to compress the GCTA dataset NA12878 (read 2) within 6 h(21,600 s, which is empirically set). On datasets SRR5419422 and ERR137269, compressed files generated by LFQC cannot be decompressed, which are considered as errors (possibly because SRR5419422 is a RNA dataset and ERR137269 is a metagenomics dataset). Table 4 reveals that the reference-based methods LW-FQZip [5] and LFQC [15] are very slow on large datasets like NA12878 (read 2). DSRC2 [12], which is the representative of reference-free methods, performs best in terms of the average compression speed. GTZ ranks second in terms of compression time.

**Table 4:** Compression time of different tools on 8 FASTQ datasets

Dataset	Size (MB)	Compression Time (s)						
		GTZ	DSRC2	QUIP	LW-FQZip	Fqzcomp	LFQC	pigz
ERR233152	556.1	19	13	10	284	13	297	**3**
SRR935126	9754.6	49	**40**	195	3966	191	3610	129
SRR489793	12,807	51	**49**	343	4893	289	4253	122
SRR801793	2756.2	43	28	59	1212	73	1143	**22**
SRR125858	50,744.2	178	**153**	1044	18,300	977	10,202	481
SRR5419422	15,094.6	26	**7**	329	4234	267	ERROR	67
ERR1137269	56,543	117	**32**	806	12,018	851	ERROR	213
NA12878 (read 2)	202,631	820	700	4703	TLE	4389	TLE	**620**
Average speed (MB/s)		267.4	**648.8**	49.7	2.9	49.6	33.7	176.8

The best results of all the tools are boldfaced

However, we are mostly interested in the total time of compression and transmission. Under the condition where the data transmission throughput is 10Gb/s (1.25 GB/s at best of AWS settings), we tested and estimated the total time of all tools and the results are listed in Table 5. To note, this is a very optimistic optimization. Here, only GTZ supports data upload while compressing, other tools have to finish compression before submission. We can see the average compression and upload speed of GTZ (269.3 MB/s) is the highest, DSRC2 comes second with an average speed of 269.1 MB/s. In general, if the input data size is very large, GTZ will be even faster than DSRC2: 7% faster in the case of the SRR125858 dataset (a 50GB dataset).

**Table 5:** Total time of different tools on 8 FASTQ datasets with maximum bandwidth

Dataset	Size (MB)	Compression Time (s) + Data best upload time						
		GTZ	DSRC2	QUIP	LW-FQZip	Fqzcomp	LFQC	pigz
ERR233152	556.1	19.0	13.4	10.4	284.4	13.4	297.4	**3.4**
SRR935126	9754.6	49.0	**48.8**	202.8	3973.8	198.8	3617.8	136.8
SRR489793	12,807	**51.0**	59.2	353.2	4903.2	299.2	4263.2	132.2
SRR801793	2756.2	43.0	30.2	61.2	1214.2	75.2	1145.2	**24.2**
SRR125858	50,744.2	**178.0**	193.6	1084.6	18,340.6	1017.6	10,242.6	521.6
SRR5419422	15,094.6	26.0	**19.1**	341.1	4246.1	279.1	ERROR	79.1
ERR1137269	56,543	117.0	**77.2**	851.2	12,063.2	896.2	ERROR	258.2
NA12878 (read 2)	202,631	820.0	862.1	4865.1	TLE	4551.1	TLE	**782.1**
Average speed (MB/s)		**269.3**	269.1	45.2	7.8	47.9	17.9	181.1

The best results of all the tools are boldfaced

To note, the upload time are estimated with the maximum bandwidth, while in practice, the upload speed could be much slower than that. To verify this, we carried out a real upload test using the relatively big dataset, SRR125858_2.fastq (about half of the SRR125858 dataset), which is 25GBs in size. The compression ratios of GTZ and DSRC2 happen to be the same on this dataset. It took GTZ 99 s to finish compression and transmission, while it took 122 s for DSRC2. Our optimistic estimation of a fast upload takes only 20.3 s, whereas in practice, it took about 45 s. The details are listed in Table 6.

**Table 6:** Total time of different tools on the SRR125858_2 dataset in a real test

Metrics	Comparative methods						
	GTZ	DSRC2	QUIP	LW-FQZip	Fqzcomp	LFQC	pigz
Compression ratio (%)	19.2	19.2	18.7	23.2	28.7	18	30.7
Total time (s)	99	122	553	9283	549	4982	324

In Table 7, we present a qualitative performance summary of all tools. The parameters, high, moderate, and low show the comparison between different tools. Compression ratio of a tool is said to be high if it is the best compressor or close to the known best algorithm. GTZ achieves satisfactory results both in compression ratio and compression speed (as well as the total time considering data upload) on tested datasets.

**Table 7:** Qualitative performance summary

Algorithm	Compression speed	Compression ratio
GTZ	High	Moderate
DSRC2	High	Moderate
QUIP	Moderate	Moderate
LW-FQZip	Low	Moderate
Fqzcomp	Moderate	Low
LFQC	Moderate	Low
pigz	High	High

### Compression rate on different data sections

The compression rates of GTZ on the three sections of a FASTQ file are reported in Table 8.

**Table 8:** The compression ratio of GTZ on the three components of FASTQ files

Dataset	Compression ratio (%)		
	Metadata	Reads	Quality scores
ERR233152	2.62	20.6	20.8
SRR935126	3.29	22.2	25.3
SRR489793	0.01	22.7	29.95
SRR801793	3.73	23.15	31.1
SRR125858	2.81	23.3	28.25
SRR5419422	0.01	22.9	9.5
ERR1137269	3.23	24.05	19.35
NA12878 (read 2)	7.59	20.4	27.3
Average	2.91	22.39	23.94

## Conclusions

The dramatic development of NGS technology has brought about challenge to store and transmit genome sequences. Efficient compression tools are feasible solutions to address this problem. Therefore, an efficient lossless compression tool for cloud computing of FASTQ files, GTZ, was proposed in this paper. GTZ is the champion winning solution of the GCTA competition (Reports can be found at http://vcbeat.net/35028.html. GTZ integrates the context modeling technology with multiple prediction modelling schemes. It also introduces the ability of paralleling processing technique for improved and steady efficiency of compression. Moreover, it enables random access to some certain specific reads. By virtue of block storage, users are allowed to only compress and read some parts of genome sequences, without the need for a complete decompression of the original FASTQ file. Another important feature is that it can overlap the data transmission with the compression process, which can greatly reduce the total time needed.

We evaluated the performance of GTZ on eight real-world FASTQ datasets and compared it with other state-of-the-art tools. Experimental results validate that GTZ performs well in terms of both compression rate and compression speed and its performance is steady across different datasets. GTZ managed to compress and transfer a 200GB FASTQ file to cloud storages like AWS and Alibaba cloud within 14 min.

For future work, we will investigate how DSRC2, which exhibits a good performance of compression alone, can be optimized for the cloud environment by utilizing data segmentation and the optimization techniques proposed in GTZ.

## Additional file

Additional file 1: Compression ratios, compression time and descriptions of datasets are included in this file. (XLSX 19 kb)
